# Epigenetic Variation May Compensate for Decreased Genetic Variation with Introductions: A Case Study Using House Sparrows (*Passer domesticus*) on Two Continents

**DOI:** 10.1155/2012/979751

**Published:** 2012-02-09

**Authors:** Aaron W. Schrey, Courtney A. C. Coon, Michael T. Grispo, Mohammed Awad, Titus Imboma, Earl D. McCoy, Henry R. Mushinsky, Christina L. Richards, Lynn B. Martin

**Affiliations:** ^1^Department of Integrative Biology, University of South Florida, SCA 110, 4202 East Fowler Avenue, Tampa, FL 33620, USA; ^2^Ornithology Section, Department of Zoology, National Museum of Kenya, Museum Hill Centre, Museum Hill Road, Westlands, Nairobi, Kenya

## Abstract

Epigenetic mechanisms impact several phenotypic traits and may be important for ecology and evolution. The introduced house sparrow (*Passer domesticus*) exhibits extensive phenotypic variation among and within populations. We screened methylation in populations from Kenya and Florida to determine if methylation varied among populations, varied with introduction history (Kenyan invasion <50 years old, Florida invasion ~150 years old), and could potentially compensate for decrease genetic variation with introductions. While recent literature has speculated on the importance of epigenetic effects for biological invasions, this is the first such study among wild vertebrates. Methylation was more frequent in Nairobi, and outlier loci suggest that populations may be differentiated. Methylation diversity was similar between populations, in spite of known lower genetic diversity in Nairobi, which suggests that epigenetic variation may compensate for decreased genetic diversity as a source of phenotypic variation during introduction. Our results suggest that methylation differences may be common among house sparrows, but research is needed to discern whether methylation impacts phenotypic variation.

## 1. Introduction

Epigenetic variation may be important to ecology [1–4], and understanding its mechanistic basis will provide insights into population processes at both ecological and evolutionary time scales [5]. Epigenetics is the study of stably heritable phenotypes that occur without alterations in the DNA sequence [6]. Molecular epigenetic modifications, such as methylation, histone deacetylation, and small interfering RNAs, regulate gene expression and are a significant contributor to phenotypic variation in diverse taxa [7]. Epigenetic modifications may vary between genome regions, over time, and in response to environmental stressors [1, 8, 9] and even among individuals and populations [10–15]. Epigenetic modification of gene expression may enable organisms to adjust their phenotypes to match novel environments or provide them the ability to quickly respond to a changing environment [16].

 Epigenetic variation potentially has an ecologically relevant role in the adaptation of introduced or invasive species to novel environments. Typically, introduced or invasive species will not be adapted to their new environments and will be hampered by reduced genetic variation associated with bottlenecks or founder effects, which creates a genetic paradox [17]. Over the ecological time scales of invasions, mutation and recombination would rarely provide sufficient sources of variation for the often extensive phenotypic differentiation that is observed among populations [4]. Epigenetic variation may be one mechanism that compensates for the lack of genetic variation in the successful introduced species, allowing the short-term adaptation to the new environment and allowing the new environment to influence genome function in the introduced species [17]. Also, if some species (or populations) are better able to regulate expression of genes via epigenetic mechanisms, which then affects the expression of ecologically important phenotypes, their ability to colonize new areas or expand their native ranges may be enhanced [3, 18–20].

The most studied molecular epigenetic mechanism is DNA methylation [21], usually of 5′methylcytosine where cytosine is followed by guanine (CpG sequences). These sequences are particularly common in gene regulatory sequences [4]. DNA methylation can cause phenotypic variation in flower shape and fruit pigmentation [22, 23], mouse tail shape, adult body size, and coat color [24, 25], and in numerous traits differentiating queen and worker honeybees [26]. DNA methylation is also known to be important in imprinting (differential gene expression depending on the parent of origin) [27], X-inactivation [28], silencing transposable elements [27], and response to environmental stressors [1, 8]. Importantly too, traits modified by DNA methylation have been stably inherited for at least eight generations [29].

DNA methylation is also a potential source of interindividual phenotypic variation [4] because of its propensity to alter gene expression contingent on environmental change [1], which could generate phenotypic variation even in cases of reduced genetic variation. There are several studies of ecological epigenetics using DNA methylation in plants. Different amounts of methylation were observed between an elite rice hybrid and its parentals [30], in phenotypically variable strains of *Brassica oleracea* [31], and among *Arabidopsis thaliana* accessions [10, 12]. The formation of the hybrid species *Spartina anglica* involved a large number of methylation changes, which as the authors noted, could play a role in the increased ecological breadth and morphological plasticity displayed by this species compared to the parental species [32]. Recently, a high level of inter-individual DNA methylation variation was detected in the violet (*Viola cazorlensis*), and variation among individuals was related to the amount of damage caused by herbivory [15]. Also, genetically identical dandelion (*Taraxacum officinale*) plants developed variation in DNA methylation in response to stressors (i.e., chemical induction of pathogen and herbivory responses), and many of these changes were stably inherited in the next generation [8]. These findings suggest that DNA methylation may provide an ecologically important source of phenotypic variation among individuals.

The house sparrow (*Passer domesticus*) is a promising organism in which to study the ecological importance of DNA methylation in wild vertebrates in response to introductions into new locations. The house sparrow has been successfully introduced throughout the world [33]. Phenotypic differentiation is extensive with populations exhibiting latitudinal and altitudinal clines in morphology, physiology, behavior, and life history characteristics in the native and introduced ranges [33–35]. Such extensive phenotypic diversification is surprising given that the short periods of time populations have had to adapt to new environments and the founder effects and/or bottlenecks that likely occurred with introductions (typically <150 years) [36].

Our study is the first yet for a wild bird species and the first to empirically investigate the role of epigenetic variation in introduced species. Relatively little information is available concerning DNA methylation in birds. However, several DNA methyltransferase enzymes and several putative DNA methyltransferase enzymes are present in the chicken genome [37], which suggests that DNA methylation is an active mechanism in birds. Our objective was to determine whether DNA methylation is variable in the house sparrow and if this variation could compensate for decreased genetic variation associated with introductions. This research is part of an ongoing effort to understand the causes of phenotypic variation among native and introduced populations [36, 38, 39]. We screened genomic CpG methylation using methylation-sensitive AFLP (MS-AFLP) among multiple individuals from Nairobi and Tampa. The MS-AFLP technique detects the methylation state of a particular recognition sequence. Thus, we were able (i) to establish if variation in DNA methylation occurs in a wild avian species, (ii) to characterize the variation that occurred among individuals and between populations, and (iii) to determine whether DNA methylation patterns differ between populations. We also could determine if DNA methylation in a wild vertebrate is similar to that observed among plants.

We screened individuals from Nairobi and Tampa because the two populations differed in time since introduction [33, 40, 41]: one introduced less than 50 years ago (Nairobi, Kenya) and one resident for about 150 years (Tampa, Florida, USA). House sparrows from these populations have different levels of genetic diversity at multiple microsatellite loci [36]. A sample from Nairobi had less genetic diversity than samples from the native European and introduced North American ranges [36], while the introduced North American populations screened, including Tampa, had similar genetic diversity as native populations. All populations screened were genetically differentiated, and the Nairobi sample was more strongly differentiated from the remaining sites, potentially because of a recent founder effect reducing genetic diversity in this area. Thus, the Nairobi sample has the genetic characteristics of a recent founder effect or bottleneck, likely associated with introduction, while the Tampa sample now has similar genetic diversity as native populations. We compared the relative amounts of epigenetic variation to genetic variation between Nairobi and Tampa. If Nairobi and Tampa have similar amounts of variation in DNA methylation, given that Nairobi has less genetic variation, it is possible that this epigenetic mechanism compensates for the decrease in genetic diversity associated with introductions as a source of phenotypic variation.

## 2. Materials and Methods

We collected house sparrow adults in Tampa, Florida, USA (*n* = 16) in spring 2008 and in Nairobi, Kenya (*n* = 14) in summer 2008. We bled individuals at capture, preserved the collected blood in a saline solution, and kept them at room temperature until DNA extraction with the DNeasy Kit (Qiagen, Valencia CA). Our objective was to determine how DNA methylation varied among individuals. DNA methylation could differ among tissues, so we used the same type of sample, blood, extracted with similar methods, for all individuals. We selected blood to match ongoing research in the Martin Lab [33–35] focused on the role of the immune and stress response in house sparrow population expansion. We performed methylation-sensitive-amplified fragment length polymorphism (MS-AFLP) following a previously described protocol [42]. For MS-AFLP, we modified an AFLP protocol [43] by substituting methylation sensitive isoschizomeric enzymes MspI and HpaII for MseI. The enzymes MspI and HpaII have different sensitivities to cytosine methylation. Thus, if the AFLP protocol is performed independently for each enzyme for each individual, the resulting banding pattern indicates the methylation state of a particular restriction site. Both enzymes cut at a CCGG restriction sequences, but *Msp*I does not cut when the inner C is methylated, while *Hpa*II does not cut when the outer or both cytosines are methylated. Together, four different types of variation can be scored [31]; Type I both enzymes cut indicating that the restriction site is not methylated, Type II *MspI* does not cut, and *HpaII* does cut indicating that the restriction site has a methylated internal C, Type III *MspI* does cut and *HpaII* does not cut indicating that the restriction site has a methylated outer C, and Type IV neither enzyme cuts indicating that either both Cs are methylated or the restriction site has mutated.

We digested DNA extracts with both EcoRI/MspI and EcoRI/HpaII enzyme combinations independently by combining approximately 200 ng DNA with 10 U of both EcoRI and MspI, and 10 U of both EcoRI and HpaII independently, in a 20 *μ*L reaction and incubated at 37°C for 6 hours (all restriction enzymes were from New England Biolabs Ipswich, MA, USA). We then ligated double-stranded EcoRI and Msp/HpaII adaptors to the digested fragments with T4 DNA ligase (New England Biolabs Ipswich, MA, USA). We conducted preselective PCR with primers designed for the EcoRI and MspI/HpaII adaptors (EcoRI 5′GACTGCGTACCAATTC; MspI/HpaII: 5′ATCATGAGTCCTGCTCGG) at a final volume of 25 *μ*L. Preselective PCR products were diluted to 100 *μ*g/*μ*L. We used one primer combination for selective PCR (MspI/HpaII: 6-FAM-CATGAGTCCTGCTCGGTCCA, EcoRI: GACTGCGTACCAATTCCGCTG). We conducted selective PCR at a final volume of 10 *μ*L; the thermal cycle was 95°C 2 m, 95°C 30 s, 53°C 30 s, 72°C 30 s, 70°C 5 m, repeated 40 times. We labeled the MspI/HpaII-selective primer with 6-FAM for visualization. We diluted PCR products 1 : 1 with loading buffer (deionized formamide, blue dextran EDTA, and MRK 500, The Gel Company San Francisco, CA, USA) and electrophoresed them on an ABI 377 (Applied Biosystems Foster City, CA, USA). We used GENESCAN 3.2.1 and GENOTYPER v 2.5 (Applied Biosystems Foster City, CA, USA) to analyze gel images and define band sizes.

We scored individuals at each enzyme combination and identified the type of epigenetic variation for each individual at each identified restriction site. We iterated the entire protocol twice for at least two individuals from each population in order to determine which restriction sites were reliably detectable. We adopted a conservative approach to scoring the gel images as AFLP-type reactions can generate variable banding among and within individuals. For a scored position to be considered reliable, the bands had to be identical and clearly distinguishable in each replicate of a given sample. Also, if subsequent reactions on additional samples generated inconsistent or unclear bands, or bands occurred at highly variable intensities at a site, that site was dropped from the analysis. We pooled data into two categories [31]: methylated (Type II and Type III) or not methylated (Type I, Type IV) restriction sites and constructed epi-haplotypes to characterize the state of DNA methylation at each site for each individual. Type IV variation at MS-AFLP could be generated either by epigenetic modification or a change in DNA sequence at the restriction site or by the gain/loss of an adjacent restriction site. Because it is not possible to accurately diagnose the underlying change, we did not include this state as methylated in our analysis.

We performed an AMOVA using GENALEX-6 [44] to calculate Φ_ST_ to characterize the amount of epi-haplotypic differentiation between Tampa and Nairobi. We conducted AMOVA over all restriction sites and independently for each restriction site. We also used BAYESCAN [45] to identify outlier loci as those potentially under selection. BAYESCAN compares a model with selection to one without selection for each restriction site. Bayes factors are calculated for each restriction site, and sites with positive Bayes factors are potentially under selection [45].

## 3. Results

Variation in DNA methylation was present among individual house sparrows (Table 1). Every individual had a unique epigenotype when all scored restriction sites were considered. We could confidently score 23 variable restriction sites from the 50 banding positions between 70 and 250 base pairs in length for both restriction enzymes. There were differences in DNA methylation among individuals at each of the 23 restriction sites. Type I (no methylation) and Type II variation occurred in certain individuals at all of the 23 restriction sites in both Nairobi and Tampa. Type III variation only occurred in certain individuals at 11 restriction sites, while Type IV variation occurred in certain individuals at 19 restrictions sites.

When the type of variation in DNA methylation was considered between locations, each type occurred in differing proportions (Table 1). Type I variation was more frequent at 14 restriction sites in Nairobi and 9 in Tampa. Type II variation was more frequent at 12 restriction sites in Nairobi and 11 in Tampa. Type III variation was in higher frequency at 5 restriction sites in Nairobi and 6 in Tampa. One restriction site had Type III variation only in Nairobi and 6 restriction sites only in Tampa. Type IV variation was more frequent at 5 restriction sites in Nairobi and at 12 sites in Tampa. One restriction site had Type IV variation only in Nairobi, and five restriction sites had Type IV variation only in Tampa.

**Figure 1:**
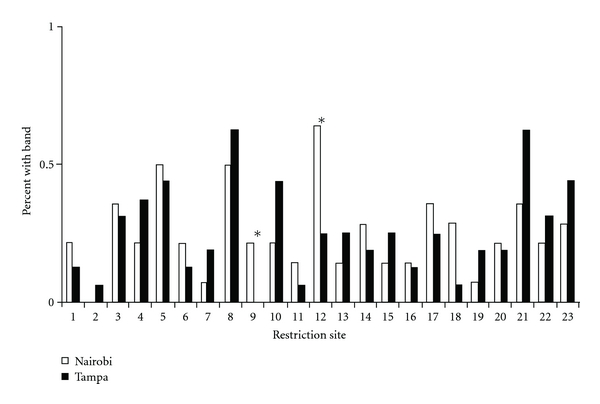
Comparison of percentage of house sparrow individuals with methylated CpG sites (Type II and Type III combined) at 23 restriction sites between Tampa (*n* = 16, black bars) and Nairobi (*n* = 14, white bars). An asterisk indicates the two outlier loci, restriction sites 9 and 12.

All restriction sites had different proportions of methylation among individuals between Nairobi and Tampa (Figure 1). Twelve restriction sites (*n* = 12) had a higher frequency of methylation in Nairobi, while 11 had a higher frequency of methylation frequency in Tampa. Restriction site 2 was only methylated in Tampa, and restriction site 9 was only methylated in Nairobi. 

The AMOVA performed over all restriction sites did not detect significant differentiation between Nairobi and Tampa (Φ_ST_ = 0.001, *P* = 0.420; Table 2). When AMOVA was calculated restriction site-by-restriction site however, two restriction sites had stronger Φ_ST_ estimates (site 9 Φ_ST_ = 0.17, *P* = 0.09; site 12 Φ_ST_ = 0.22, *P* = 0.06). The two restriction sites had higher proportions of methylation in Nairobi (Figure 1, sites 9 and 12). BAYESCAN also identified these two loci as the strongest outliers (Site 9 Bayes Factor = 0.04; Site 12 Bayes Factor = 0.03); however, the Bayes Factors were relatively weak. 

## 4. Discussion

Epigenetic variation, in the form of DNA methylation at CpG sites, occurred in wild house sparrows. This variation could be screened with a simple and reliable MS-AFLP technique. A great deal of variation occurred among individuals, and all screened individuals had unique epigenotypes. All types of methylation were present in both populations, indicating that both epigenetic and genetic variation (indicated by Type IV variation) exists within and between Nairobi and Tampa populations. We observed more methylation overall in the Nairobi population; however, some restriction sites were more methylated in Tampa, and most restriction sites had different frequencies of methylation between Nairobi and Tampa. Nairobi and Tampa had more similar levels of variation in DNA methylation than at microsatellite loci, where Nairobi had fewer alleles, lower heterozygosity, and more private alleles than Tampa [36]. Thus, it is possible that epigenetic variation may provide a source for the phenotypic diversity found in the more recently introduced populations and compensate for the decreased genetic variation.

When the frequency of methylation was compared between populations at all restriction sites, no significant difference was detected, and the amount of within-population variation was much greater than the between-population variation. Given the amount of variation detected and that the state of DNA methylation could change in opposing ways at each site (i.e., from methylated to nonmethylated at one site, yet from unmethylated to methylated at another), our results have suggested it would take a great deal of statistical power to detect differences among all restriction sties. However, we identified two restriction sites with a stronger signal differentiating Nairobi and Tampa, and these sites approached statistical significance, indicating that screening additional MS-AFLP selective primer combination could identify sufficient restriction sites to discriminate locations. These findings suggest that there may be a complicated relationship among variable restriction sites and that only a few of the variable sites may be ecologically important (sensu [14]). Thus, the amount of within-population variation observed across presumably mostly neutral loci was so great that we were not able to detect an overall signal of differentiation between populations. As in the recent study of* V. cazorlensis *populations [14, 15], our results suggest that MS-AFLP data may require attention to detect atypical outlier loci, which are important for a particular trait, yet are only a subset among several variable restriction sites. Our analysis suggests that it would be very difficult to detect differences between populations without scanning for outliers.

It is possible that in some cases the differences between MspI/EcoRI and HpaII/EcoRI reactions could have been generated by inconsistent restriction digests rather than differential methylation. However, inconstant digestion could generate false methylated or false unmethylated results, and we performed a long restriction digestion (6 h) and used a conservative scoring approach to minimize potential errors. Thus, given the high level of variation detected, the main conclusions of this study would not be affected greatly by a modest error rate in restriction digests.

Substantial phenotypic divergence has occurred among introduced house sparrows within 150 years [33–35]. Epigenetic variation is a potential mediator of rapid evolution of introduced species to new environments [17] and has been linked to phenotypic variation [4, 46]. Together, the presence of MS-AFLP variation in house sparrows and the persistence of latitudinal patterns of phenotypic variation among introduced populations predominantly coming from western European sources [35] support the possible role of epigenetic variation as a mediator of phenotypic diversity in introduced populations.

## 5. Conclusions

Given the amount of variation observed, it is plausible that epigenetic variation may compensate for decreased genetic variation as a source of phenotypic variation within introductions. It is also plausible that epigenetic variation may be responsible for some of the phenotypic differentiation among individuals. However, the MS-AFLP technique alone does not allow for the identification of the specific underlying genetic elements that are methylated, nor does it identify the effects (i.e., silencing or enhancing) on gene expression. Also, DNA methylation likely has multiple functions in addition to the possible role in phenotypic differentiation (e.g., silencing transposable elements). Thus, it may be that only a small subset of fragments screened with the MS-AFLP technique may regulate expression of genes determining phenotypic traits. Also, we screened DNA methylation in blood samples, and additional variation in DNA methylation may occur in other tissues, and the variation in other tissues could occur in different patterns. However, the level of variation detected in blood suggests that DNA methylation would be variable in other tissues. Additional research will be critical to characterize epigenetic variation at restriction sites that are functionally related to phenotypic differences, but presently only a few examples exist in which gene methylation has demonstrable phenotypic effects in animals [47]. Our labs are presently investigating the effects of methylation of the glucocorticoid receptor promoter on brain and behavior (sensu [48]), and the present study demonstrates that such an effort could be fruitful in house sparrows.

Also, as this study is a two-population comparison, it is premature to conclude that differential methylation is pervasive among sparrow populations. However, the extensive phenotypic variation despite moderate genetic differentiation that exists among populations of introduced house sparrows [36] indicates that epigenetic modification could be important. Simple next steps to assess the relevance of methylation would entail comparisons (i) among species with different levels of introduction success, (ii) populations at the edges and centers of ranges, or (iii) populations that differ in time since introduction/colonization. It may also be informative to characterize the methylation present in different tissues at different times after a stimulus (i.e., a stressor or immune challenge), as this approach could implicate the restriction sites and hence the genes that contribute directly to phenotypic variability.

## Figures and Tables

**Table 1:** Frequency of epigenetic variation detected by MS-AFLP at the restriction site CCGG. The type of epigenetic variation is presented following Salmon et al. [31]; Type I = restriction site no methylation, Type II = methylation of internal C, Type III = methylation of external C, and Type IV = hypermethylation or mutation in restriction site.

	Restriction site																						
	1	2	3	4	5	6	7	8	9	10	11	12	13	14	15	16	17	18	19	20	21	22	23
Nairobi																							
Type I	0.79	1	0	0.64	0.21	0.79	0.93	0.29	0.64	0.71	0.86	0.29	0.21	0.43	0.86	0.5	0.5	0.07	0.86	0.71	0.64	0.79	0.71
Type II	0.21	0	0.21	0.14	0.5	0.21	0.07	0.5	0.21	0.14	0.14	0.64	0.14	0.29	0.14	0.14	0.36	0.21	0.07	0.21	0.36	0.21	0.14
Type III	0	0	0.14	0.07	0	0	0	0	0	0.07	0	0	0	0	0	0	0	0.07	0	0	0	0	0.14
Type IV	0	0	0.64	0.14	0.29	0	0	0.21	0.14	0.07	0	0.07	0.64	0.29	0	0.36	0.14	0.64	0.07	0.07	0	0	0

Tampa																							
Type I	0.88	0.94	0.38	0.31	0.5	0.88	0.75	0.25	0.75	0.44	0.88	0.5	0.19	0.13	0.63	0.69	0.63	0	0.81	0.63	0.31	0.69	0.44
Type II	0.13	0.06	0.19	0.38	0.38	0.06	0.19	0.63	0	0.38	0.06	0.25	0.19	0.19	0.25	0.06	0.25	0.06	0.13	0.13	0.63	0.31	0.38
Type III	0	0	0.13	0	0.06	0.06	0	0	0	0.06	0	0	0.06	0	0	0.06	0	0	0.06	0.06	0	0	0.06
Type IV	0	0	0.31	0.31	0.06	0	0.06	0.13	0.25	0.13	0.06	0.25	0.56	0.69	0.13	0.19	0.13	0.94	0	0.19	0.06	0	0.13

**Table 2:** Summary AMOVA table for the comparison among all sites between house sparrows from Florida and Kenya (d.f.: degrees of freedom).

Source	d.f.	Sum of squares	Mean square	Estimated variance
Among populations	1	4.107	4.107	0.004
Within populations	28	113.393	4.050	4.050
Total	29	117.500		4.054
Φ_ST_	0.001			
*P*	0.420			
